# An Orthologue of the Retinoic Acid Receptor (RAR) Is Present in the Ecdysozoa Phylum Priapulida

**DOI:** 10.3390/genes10120985

**Published:** 2019-11-29

**Authors:** Elza S. S. Fonseca, Youhei Hiromori, Yoshifumi Kaite, Raquel Ruivo, João N. Franco, Tsuyoshi Nakanishi, Miguel M. Santos, L. Filipe C. Castro

**Affiliations:** 1 CIIMAR/CIMAR Interdisciplinary Centre of Marine and Environmental Research, U.Porto, 4450-208 Matosinhos, Portugal; fonseca.ess@gmail.com (E.S.S.F.); ruivo.raquel@gmail.com (R.R.); joaonunofranco@gmail.com (J.N.F.); 2 FCUP—Faculty of Sciences, Department of Biology, U.Porto, 4169-007 Porto, Portugal; 3 Laboratory of Hygienic Chemistry and Molecular Toxicology, Gifu Pharmaceutical University, Gifu 501-1196, Japan; hayake5@hotmail.com (Y.H.); 135018@gifu-pu.ac.jp (Y.K.); 4 Faculty of Pharmaceutical Sciences, Suzuka University of Medical Science, Suzuka 513-8670, Japan

**Keywords:** Bilateria, nuclear receptors, xenobiotics, endocrine system

## Abstract

Signalling molecules and their cognate receptors are central components of the Metazoa endocrine system. Defining their presence or absence in extant animal lineages is critical to accurately devise evolutionary patterns, physiological shifts and the impact of endocrine disrupting chemicals. Here, we address the evolution of retinoic acid (RA) signalling in the Priapulida worm, *Priapulus caudatus* Lamarck, 1816, an Ecdysozoa. RA signalling has been shown to be central to chordate endocrine homeostasis, participating in multiple developmental and physiological processes. Priapulids, with their slow rate of molecular evolution and phylogenetic position, represent a key taxon to investigate the early phases of Ecdysozoa evolution. By exploring a draft genome assembly, we show, by means of phylogenetics and functional assays, that an orthologue of the nuclear receptor retinoic acid receptor (RAR) subfamily, a central mediator of RA signalling, is present in Ecdysozoa, contrary to previous perception. We further demonstrate that the Priapulida RAR displays low-affinity for retinoids (similar to annelids), and is not responsive to common endocrine disruptors acting via RAR. Our findings provide a timeline for RA signalling evolution in the Bilateria and give support to the hypothesis that the increase in RA affinity towards RAR is a late acquisition in the evolution of the Metazoa.

## 1. Introduction

**Figure 1:**
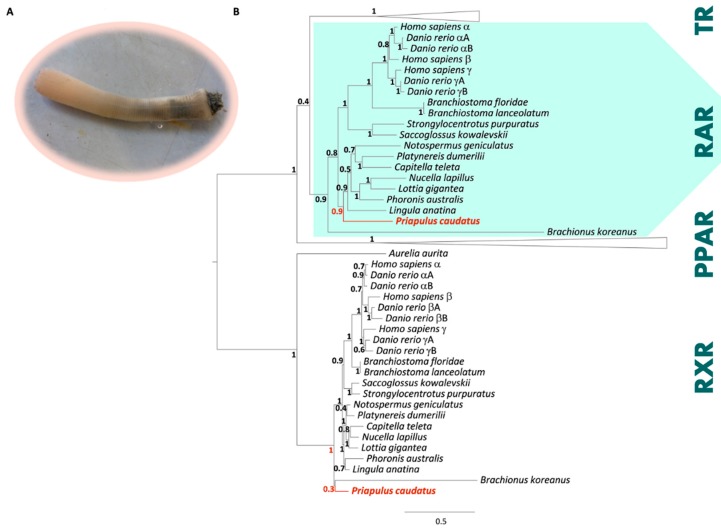
The retinoic acid receptor (RAR) and retinoid X receptor (RXR) nuclear receptors of *P. caudatus*. (**A**) A specimen photograph of *P. caudatus*. (**B**) Maximum likelihood phylogenetic tree of RAR, RXR, thyroid hormone receptor (TR) and peroxisome proliferator-activated receptor (PPAR) nuclear receptors; numbers at nodes indicate posterior probabilities calculated using aBayes. Photograph by João N. Franco.

Retinoic acid (RA) is a critical regulator of multiple biological processes in vertebrates including cell differentiation and embryonic development [1,2], central nervous system development [3,4,5], organ formation and tissue maintenance [6,7,8] and vision [9]. Retinoids, such all-*trans* retinoic acid (ATRA), 9-*cis* retinoic acid (9cisRA) and 13-*cis* retinoic acid (13cisRA) are active metabolites of vitamin A (retinol), known to bind and modulate the retinoic acid receptor (RAR) and retinoid X receptor (RXR), the central mediators of RA signalling [10]. RAR and RXR belong to the nuclear receptor (NR) superfamily and are ligand-dependent transcription factors that regulate the expression of specific genes [11,12]. RAR heterodimerizes with RXR and recognizes specific RA responsive elements (RAREs) in the regulatory region of the target gene [13,14]. Upon binding to ligands, the position of the helix 12 on the ligand-binding domain (LBD) is modified, allowing the recruitment of coactivators and consequently, the activation of gene transcription [11,13,14]. The emergence of various non-chordate genome sequences established that RA signalling is not chordate-specific, since signalling components such as *RAR* and *RXR* gene orthologues have been found in species from Ambulacraria (echinoderms and hemichordates) [15,16,17,18,19,20]. Recently, RAR was also functionally characterized in various mollusc species and in a second Lophotrochozoa clade, the annelid worm *Platynereis dumerilii*. Yet, functional studies in mollusc species demonstrated the loss of RA binding affinity towards RAR [21,22,23,24]. In contrast, the annelid *Platynereis RAR* orthologue showed a conserved capacity to bind and respond to retinoids, but with lower affinity compared to vertebrate RAR paralogues [25]. Furthermore, it was demonstrated that RAs trigger neuronal differentiation, a role previously described only in chordates [4,21,26]. Surprisingly, in a second annelid species, *Helobdella robusta* (leech), *RAR* and other RA signaling components are absent [15]. The search for *RAR* gene orthologues in Ecdysozoa (e.g., arthropods and nematodes) genomes was previously unsuccessful, implying that *RAR* was probably lost in this superphylum [15]. Overall, these studies suggest that 1) *RAR* evolution was shaped by events of secondary gene loss during Bilateria evolution, notably in the whole Ecdysozoa lineage and Appendicularia (Tunicata) [15,16,18] and 2) the bilaterian *RAR* ancestor was a RA low-affinity sensor, with the ability to bind retinoids and activate transcription of target genes—annelids (*Platynereis*), or a receptor without capacity to bind ligands as seen in molluscs [22,23,24,25]. Additionally, NRs are prime targets of endocrine disrupting chemicals (EDCs) (e.g., [27]), with various examples denoting the impact of EDCs acting via NRs [28,29]. Yet, variations in NR gene complement and sequence variation as well as the molecular architecture of endocrine systems are of paramount importance to recognize the mechanisms of action of EDCs [30,31], particularly in invertebrate lineages, e.g., [32,33]. In the specific case of *RAR*, environmental contaminants such as pesticides have been shown to exploit mammalian *RAR*s [34], but not the molluscan orthologue [24]. To further scrutinize the evolution RA signalling, specifically if *RAR* is absent or present in other extant Ecdysozoa lineages, we investigated the genome of the penis worm, *Priapulus caudatus* Lamarck, 1816 (Scalidophora, Priapulida). Priapulids are mud-dwelling, carnivorous marine worms with a tubular body shape and an eversible proboscis (Figure 1A; see Video S1) that altered little since the arthropod/priapulid common ancestor (over 520 million years ago) [35,36,37]. Their morphological and developmental characteristics together with their slow rate of molecular evolution suggest Priapulida as a key phylum to understand the evolution of Ecdysozoa.

## 2. Materials and Methods

### 2.1. Sampling

One adult and two juvenile specimens of *P. caudatus* were collected at the Gullmarn fjord, Sweden, and preserved on RNA later (Invitrogen, Carlsbad, CA, USA) for further total RNA extraction.

### 2.2. RNA Extraction

The adult priapulid was dissected into small portions and homogenized with PureZOL RNA Isolation Reagent^®^ (Bio-Rad, Hercules, CA, USA). The extraction of nucleic acids was performed with chloroform according to the manufacturer’s instructions and the resulting aqueous phase was used to isolate the total RNA. The illustra RNAspin Mini RNA Isolation (GE Healthcare, Chicago, IL, USA) kit was used for total RNA isolation. A step of on-column DNAse I digestion was included to exclude genomic contamination and the RNA was eluted with RNase-free water, starting from the ethanol step. The iScript™cDNA Synthesis Kit (Bio-Rad, Hercules, CA, USA) was used for cDNA synthesis and performed according to the manufacturer’s recommendations, using 1000 ng of the RNA previously isolated.

### 2.3. RXR and RAR Gene Isolation

A BLAST approach conducted on the publicly available *P. caudatus* draft genome (GCA_000485595.2, Priapulus_caudatus-5.0.1) to investigate the presence of *RAR* and *RXR*-like sequences. The open reading frame (ORF) of *P. caudatus RAR* and *RXR* were deduced from the genome assembly and isolated using a polymerase chain reaction (PCR) with specific primers (Table S1). In the case of *RXR*, two partial nucleotide sequences were used to design specific primers (Table S1) and a partial *P. caudatus RXR* containing the termination codon was isolated by PCR. To obtain the remaining sequence, the partial *RXR* isolated sequence was extended using the SMARTer™ RACE cDNA Amplification Kit (Clontech, Mountain View, CA, USA) following the manufacturer instructions, using specific RACE PCR primers (Table S1). The Phusion Flash High-Fidelity PCR Master Mix (ThermoFisher, Waltham, MA, USA) was used in all PCR reactions and the obtained products were purified with NZYGelpure (Nzytech, Lisbon, Portugal), cloned into pGEM-T Easy Vector System (Promega, Madison, WI, USA). The sequences were confirmed by automated Sanger sequencing (Eurofins GATC). *RAR* and *RXR P. caudatus* sequences have been deposited in GenBank (Accession numbers MK780070 and MK780071, respectively).

### 2.4. Sequence and Phylogenetic Analysis

Amino acid sequences of RAR, RXR, thyroid hormone receptor (TR) and peroxisome proliferator-activated receptor (PPAR) from various Metazoa taxonomic groups were recovered through BLASTp searches in GenBank, Joint Genome Institute (JGI) Genome Portal and Okinawa Institute of Science and Technology (OIST) Marine Genomics Unit Genome Browser. Retrieved sequences and corresponding protein accession numbers are listed in the Supplementary material Table S2. The collected sequences were aligned with the Multiple Alignment using Fast Fourier Transform (MAFFT) programme v7 (L-INS-i method) [38], visualized and edited in Geneious ^®^v7.1.7. Based on previous studies [25,39,40,41,42], the amino acids residues that interact with ATRA were identified. The columns containing gaps were stripped, resulting in a final alignment contained 71 sequences and 277 positions. A Maximum Likelihood phylogenetic analysis was performed using the PhyML 3.0 server with the amino acid substitution model LG + G + I and the evolutionary model automatically selected [43]. The branch support for phylogenetic trees was calculated using aBayes. FigTree v1.3.1 was used to visualize the tree.

### 2.5. Construction of Plasmid Vectors

The hinge region and LBD of *RAR* and *RXR* were isolated from human and penis worm by PCR with specific primers (Table S3) and cloned into pBIND and/or pACT vectors (Promega, accession numbers AF264722 and AF264723.1), to produce “chimeric” receptors with the yeast transcriptional activator GAL4 (RAR-LBD-GAL4) or the viral enhancer, VP16 (RXR-LBD-VP16), which acts on proximal downstream promoters, respectively [44,45]. The priapulid RAR LBD was amplified by PCR from pGEM-pCauRAR with specific primer (FP: 5′-ACTGGATCCTCGATTATGTCTATGCAACAGCGA-3′, RP: 5′-GATTCTAGAACTAGTGATTTCACGGTATGCAG-3′) and the product was digested with BamHI and XbaI. The digested fragment was subcloned into BamHI–XbaI site of pCold-TF vector (TAKARA bio, accession number AB213654), for the priapulid RAR LBD−His6-tagged trigger factor hybrid protein. Plasmid sequences were confirmed using automated Sanger sequencing (Eurofins GATC). The human RARα LBD was previously cloned into pGEX-4T-1 vector (GE Healthcare Life Sciences, accession number U13853), for the human RARα LBD-Glutathione S-transferase hybrid protein [22].

### 2.6. Chemicals and Solutions

ATRA, 9cisRA, 13cisRA, endrin, dieldrin, and sterile Dimethyl sulfoxide (DMSO) were purchased from Sigma-Aldrich (St. Louis, MO, USA). The stock solutions were prepared in DMSO: ATRA, 9cisRA and 13cisRA at 0.1, 1 and 10 mM, endrin and dieldrin at 10 mM.

### 2.7. Cell Culture and Transactivation Assays

Cos-1 cells were maintained in Dulbecco’s Modified Eagle Medium (DMEM) (PAN-Biotech, Aidenbach, Bayern, Germany). A supplementation with 10% fetal bovine serum (PAN-Biotech, Aidenbach, Bayern, Germany) and 1% penicillin/streptomycin (PAN-Biotech, Aidenbach, Bayern, Germany)) was used. Cells were maintained at 37 °C with a humidified atmosphere and 5% CO_2_. Cells were seeded in 24-well culture plates at a density of 2 × 10^5^ live cells/well and after 24 h, transfected with 0.5 μg of pBIND constructs (pBIND-PcauRAR-LBD or pBIND-HsaRARγ-LBD (positive control)) and 1 μg of pGL4.31 luciferase reporter vector (DQ487213; Promega), containing five upstream activation sequence (UAS) elements, upstream of the firefly luciferase reporter gene or, in the case of heterodimer transfection assays, with 0.5 μg of pBIND constructs (pBIND-PcauRAR-LBD or pBIND-HsaRARγ-LBD), 0.5 μg pACT constructs (pACT-PcauRXR-LBD or pACT-HsaRXRγ-LBD (positive control)) and 0.5 μg of pGL4.31, using lipofectamine 2000 reagent (Invitrogen) in Opti-MEM (Gibco, Carlsbad, CA, USA), to a final volume of 350 μL. After 5 h of incubation, transfection media was replaced by DMEM phenol-free supplemented with 10% dextran-coated charcoal-treated serum (Invitrogen) and 1% penicillin/streptomycin (Invitrogen) containing the test compounds. Final concentrations were the following: 0.1, 1 and 10 μM ATRA, 9cisRA or 13cisRA and 10 μM organochlorine pesticides (endrin and dieldrin) dissolved in DMSO (0.1%). Cells were lysed 24 h after transfection. Firefly luciferase (reporter pGL4.31) and *Renilla* luciferase (pBIND) activities were assayed with Dual-Luciferase Reporter Assay System (Promega, Madison, WI, USA), according to the manufacturer’s instructions. Two technical replicates per condition in three independent assays were performed for all transfections. The results were expressed as fold-induction resulting from the ratio between luciferase (reporter pGL4.31) and *Renilla* (internal control for transfection efficiency luminescent activity), and then normalized by the DMSO control. Transactivation data was calculated as means of the normalized values (*n* = 3) and the bars with standard error of the mean (SEM) from the three separate experiments. The means of the technical replicates were used for statistical analysis with one-way ANOVA, followed by Holm-Sidak method in the SigmaPlot software v11.0. Data were transformed whenever the normality failed. The level of significance was set to 0.05.

### 2.8. Ligand Binding Assays

The PcauRAR LBD−His6-tagged trigger factor hybrid protein was expressed in *Escherichia coli* BL21 (DE3) cells containing the chaperon plasmid pG-Tf2 (Takara bio, Kusatsu, Shiga, Japan) and purified by using His-select nickel affinity gel (Sigma−Aldrich, St. Louis, MO, USA). The HsaRARα LBD-Glutathione S-transferase hybrid protein was expressed in *Escherichia coli* BL21 (DE3) cells and purified by Glutathione Sepharose 4B (GE Healthcare, Chicago, IL, USA) (positive control). Ligand binding assay was assessed as previously described [22,46,47,48]. In brief, the purified protein (12.5 μg/mL) was incubated with 10 nM of all-trans retinoic acid (11, 12-^3^H) ([^3^H] ATRA; 1.665 TBq/mmol; Amersham Biosciences) or 10 nM of Retinoic Acid, (11, 12-^3^H) 9-cis ([^3^H] 9cisRA; 1.95 TBq/mmol; PerkinElmer). Unlabeled ATRA and 9cisRA were used to compete for [^3^H] ATRA or [^3^H] 9cisRA in this assay to determine the binding preferences of PcauRAR LBD or HsaRARα LBD. Hydroxyapatite was added to precipitate the receptor protein and bound radioactive compounds after an incubation step at 4 °C for 1 h. The hydroxyapatite pellet was washed. The radioactivity in the pellet was determined by liquid scintillation counting.

## 3. Results

### 3.1. Phylogenetic and Sequence Analysis of Priapulid RAR

By thoroughly examining a genome draft of *P. caudatus*, we established the presence of sequences with high similarity to *RAR* and *RXR* respectively. Since *RAR* sequences are absent from previously analysed Ecdysozoa genomes, we went on to validate this initial screening. We experimentally isolated the full-length sequences of both *RAR* and *RXR* from *P. caudatus* cDNA. These encode two protein sequences with 491 (RAR) and 404 (RXR) amino acids. To determine the orthology of the isolated sequence a phylogenetic analysis was conducted (Figure 1B), including RAR, RXR, TR and PPAR amino acid sequences of vertebrates (human and zebrafish), cephalochordates (Florida and European lancelet), ambulacrarians (acorn worm and purple sea urchin), molluscs (Atlantic dogwhelk and owl limpet), annelids (Dumeril’s clam worm and polychaete worm), a nemertean (ribbon worm), a phoronid (phoronid worm), a brachiopod (common Oriental lamp shell), a rotifer (Korean monogonont rotifer), and a cnidarian (moon jelly).

**Figure 2:**
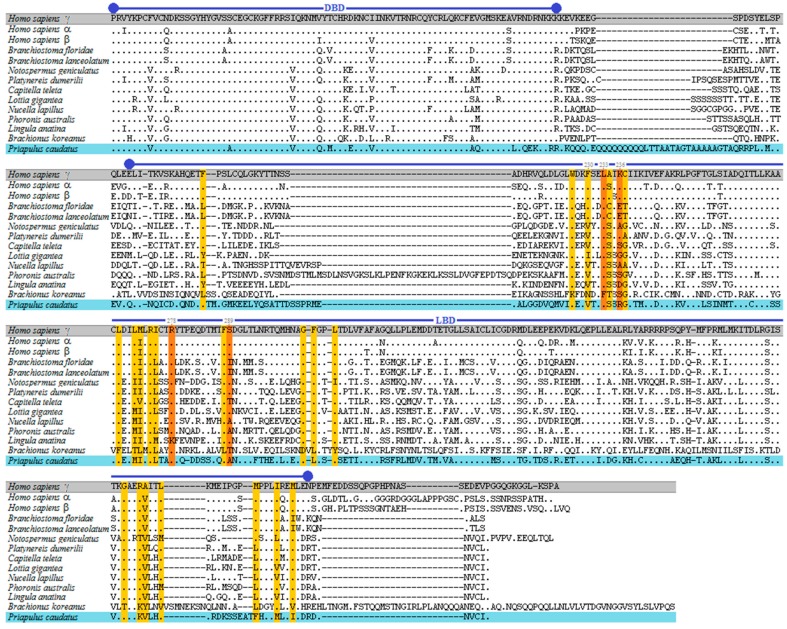
Amino acid sequence alignment of the RAR DNA- and ligand- binding domains from human, lancelets, molluscs, annelids, nemertean, brachiopod, phoronid, rotifer and priapulid RAR protein sequences. Key amino acid residues that interact with ATRA in the human RARγ ligand-binging pocket (LBP) are highlighted: orange—direct or indirect hydrogen bonds, yellow—hydrophobic and Van der Waals interactions [25,39,40,41,42]. The DBD and LBD are delimited by the upper blue lines.

The predicted *RAR* (PcauRAR) and *RXR* (PcauRXR) sequences of the priapulid worm robustly clustered in the respective clade (Figure 1B). Next, we examined the amino acid sequence alignment with PcauRAR (Figure 2), which revealed that, similarly to other RARs, PcauRAR has a conserved modular structure typical of NRs, with a conserved DNA- binding domain (DBD) and a moderately conserved LBD [49]. The PcauRAR-DBD shares approximately 80–82% of sequence identity with human and molluscs RARs, 84% with annelids RARs, 81–87% with nemertean, brachiopod and phoronid RARs, and 78% with rotifer RAR. Regarding the PcauRAR-LBD, the identity with human RARs decreases to 39–41%, with molluscs RARs to 47–49%, with annelids, nemertean and brachiopod RARs to 49–54%, and with phoronid and rotifer RARs to only 33% and 20% respectively (Figure 2).

Analysis of the key amino acid residues known to interact with ATRA in human RARγ (Hsa RARγ) ligand binding pocket (LBP) [39,42], showed that 14 out of 25 are different (Figure 2), a feature also observed in previous studies: 9 to 11 amino acids in mollusc [24] and 8 in annelids [25]. In nemertean 12 amino acids are not conserved, and, similarly to molluscs, 9 amino acids are not conserved in brachiopod and phoronid. In rotifer only 3 of the key 25 amino acids are conserved. Regarding these 25 amino acids residues, 4 (Leu233, Lys236, Arg278, and Ser289) participate in stable hydrogen bonds with the carboxyl group of ATRA in HsaRARγ. In PcauRAR, as well as, in phoronid and brachiopod RARs, two of these residues are not conserved (Lys236>Arg, Ser, Asp in priapulid, phoronid, brachiopod; Arg278>Lys in brachiopod; and Ser289>Ala in priapulid and phoronid), and only one in nemertean RAR (Lys236>Arg), whereas in the annelid RAR they are all conserved (Figure 2). Furthermore, the Phe230 residue which was demonstrated to play a crucial role for RA binding, enabling transactivation properties [39], is replaced by a Val residue among lophotrochozoans and priapulid. The mutation to a Phe in *Platynereis* results on a decreased ability of the annelid RAR to activate transcription upon binding to ATRA [25], suggesting a similar consequence for priapulid.

### 3.2. Functional Characterization of the Priapulid RAR Orthologue

**Figure 3:**
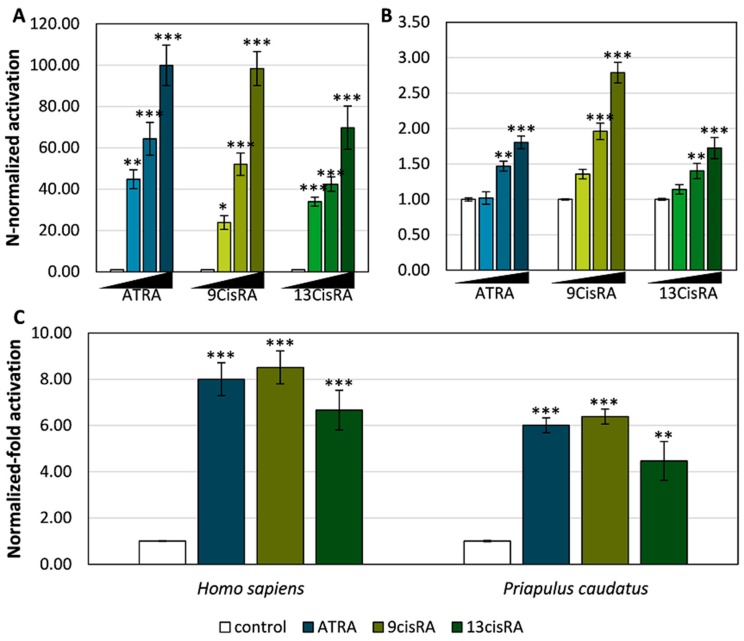
Luciferase transcription transactivation mediated by chimeric receptors in the presence of various ligands (ATRA, 9cisRA or 13cisRA at a final concentration of 0.1, 1 and 10 μM). (**A**) Human RAR-LBD-GAL4; (**B**) Priapulid RAR-LBD-GAL4; (**C**) Human and priapulid RAR/RXR heterodimers. Data represent means ± SEM from three separate experiments (*n* = 3). The results were normalized to the control condition (DMSO without ligand). Significant differences between the tested concentrations and the solvent control were inferred using one-way ANOVA. Asterisks denote significant differences (* *P* < 0.05, ** *P* < 0.01, *** *P* < 0.001) between the tested compound and the control.

**Figure 4:**
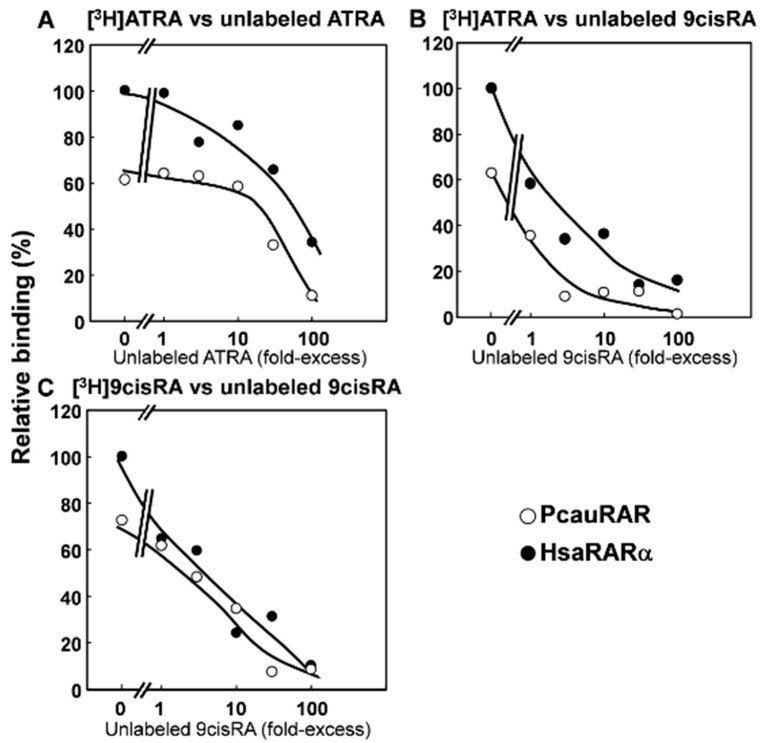
Competition by ATRA and 9cisRA with [^3^H]ATRA and [^3^H]9cisRA for binding to the LBD of PcauRAR and HsaRARα. The LBD of PcauRAR (○) and HsaRARα protein (●) was incubated with increasing concentrations of unlabeled ATRA (**A**) or 9cisRA (**B**,**C**) as competitors in the presence of [^3^H]ATRA (**A**,**B**) or [^3^H]9cisRA (**C**) as ligand. Specific binding of the radio ligands was defined as total binding minus that occurring in the presence of 1000-fold molar excess of unlabeled ATRA (**A**,**B**) or 9cisRA (**C**). Results were expressed as percentage of specific binding of the radio ligands. The binding of each radio ligand to HsaRARα in the absence of unlabeled competitors was set at 100%. Each experiment was performed at least twice, and representative curves are shown.

To unfold the binding properties of PcauRAR and compare with both mollusc and annelid RARs, we next investigated the binding profile of the priapulid orthologue to transactivate target gene transcription, performing transactivation assays with GAL4-LBD chimeric receptors. Thus, we tested the ability of PcauRAR-LBD-GAL4 to bind retinoids (ATRA, 9cisRA and 13cisRA) and to activate a luciferase reporter gene (Figure 3). Our results show that PcauRAR is able to significantly (*P* < 0.05) activate transcription upon binding to retinoids at concentrations of 1 and 10 μM (Figure 3B), but at a lesser degree than HsaRARγ (Figure 3A). The results obtained with transactivation assays were next confirmed by a competitive ligand binding assay. The ability of PcauRAR to bind to ATRA and 9cisRA was clearly demonstrated (Figure 4).

In vertebrates, RAR dimerizes with RXR [13,14,50]. Thus, we next assayed the capacity of RAR to transactivate luciferase transcription as a heterodimer with RXR, using a two-hybrid protein-protein interaction strategy (pBind/pACT system). The interaction between the chimeric proteins (RAR-LBD-GAL4 and RXR-LBD-VP16) was first verified (Supplementary material Figure S1) and then, the activation of the heterodimer was tested with ATRA, 9cisRA and 13cisRA at 10 μM (Figure 3C). As predicted, the RAR/RXR heterodimer activates luciferase transcription upon binding to the tested retinoids (*P* < 0.05) in both human and priapulid (Figure 3C).

### 3.3. Pesticides Do Not Activate Transcription via the Priapulid RAR

**Figure 5:**
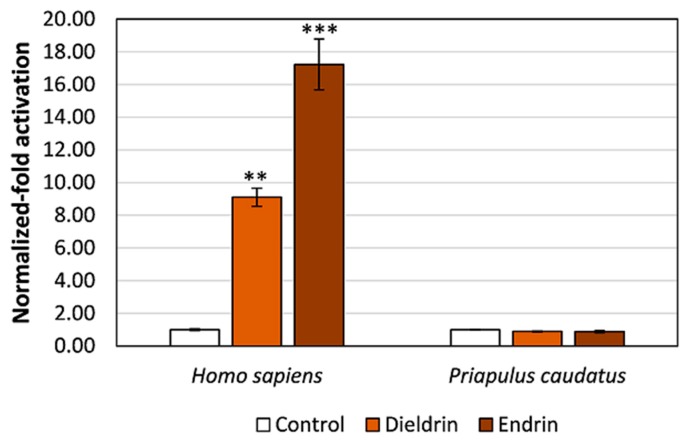
Luciferase transcription transactivation mediated by human and priapulid chimeric receptors (RAR-LBD-GAL4) in the presence of 10 μM endrin or dieldrin (organochlorine pesticides). Data represent means ± SEM from three separate experiments (*n* = 3). The results were normalized to the control condition (DMSO without ligand). Significant differences between the tested concentrations and the solvent control were inferred using one-way ANOVA. Asterisks denote significant differences (** *P* < 0.01, *** *P* < 0.001) between the tested compound and the control.

NRs are classical targets of endocrine disrupting chemicals [51,52,53]. RARs in particular have been shown to bind and activate transcription in the presence of specific toxicants [34,54]. To address whether two organochlorine pesticides (endrin and dieldrin) known as endocrine disruptive chemicals (EDCs) acting via human RARs are also binding to PcauRAR, we performed transactivation assays. Importantly, these pesticides are persistent in fishes and sediments from the Baltic Sea, where *P. caudatus* occurs [55,56,57,58]. As previously shown with mollusc RARs [24], these EDCs were unable to promote luciferase transcription through PcauRAR activation (*P* > 0.05) (Figure 5).

## 4. Discussion

**Figure 6:**
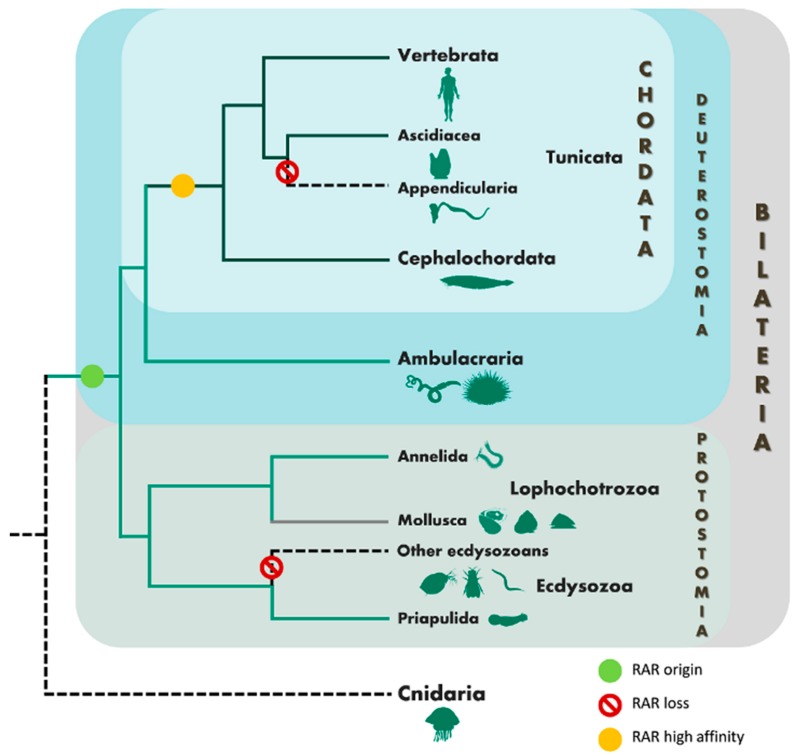
The evolution of RAR in Metazoa lineages. Black dashed lines represent no RAR known, light green full lines represent RA low-affinity sensors, dark green full lines represent RA high-affinity receptors, grey full line represent RA unresponsive sensors.

The emergence of non-chordate sequenced genomes has significantly modified the evolutionary consensus of RA signalling as a chordate-specific feature. In effect, *RAR* and other RA signalling components were described in non-chordate metazoans, such as ambulacrarians (echinoderms and hemichordates) [16] and lophotrochozoans (molluscs and annelids) [21,22,23,24,25]. Strikingly, we establish that a retinoid-activated *RAR* was retained in the Ecdysozoa *P. caudatus*. Our findings strongly support earlier studies, suggesting that *RAR* originated in the Bilateria ancestor [22,24,25], and substantiate the likely loss of this transcription factor in most lineages leading to extant Ecdysozoa species examined so far (Figure 6). Together, these results emphasize the importance of Priapulida to decipher Ecdysozoa evolution, in particular that of NR biology [35,59].

By inspecting a RAR protein sequence alignment, we show that the penis worm orthologue exhibits the characteristic modular structure of NRs and displays a higher sequence homology with annelid RARs than with mollusc and vertebrate RARs. The retinoid binding profile of PcauRAR was corroborated with both transactivation assays and a competitive ligand binding assay that clearly established the ability of PcauRAR to bind to ATRA and 9cisRA, as it had been previously demonstrated with the annelid *RAR* [25], but not in molluscs [22,23,24]. Yet, our findings consistently show that PcauRAR exhibits a low affinity for the tested ligands (retinoids)—in the micromolar range, similar to previous findings for the *Platynereis RAR* orthologue [25]. This is in stark contrast with the affinity shown by chordate orthologues (nanomolar scale). In effect, the operating mode of PcauRAR in the presence of retinoids (ATRA, 9cisRA and 13cisRA) significantly induced luciferase transcription via PcauRAR activation, but at lower levels than HsaRARγ, as suggested by the LBP composition. Previous studies with crystallographic analysis of human RARγ [39] and *Platynereis* RAR [25] in complex with ATRA revealed a strong divergence in the structural interaction on how ATRA binds the RAR LBP residues in these species. In human RARs, 25 amino acid residues are crucial for the interaction with ATRA, with 4 of these residues forming direct or indirect hydrogen bonds with the carboxyl group of retinoids [39,42]. Despite the conservation of these 4 residues, the interaction of retinoids with annelid RAR-LBP is dominated by loose van der Waals forces and no hydrogen bond with retinoid carboxyl group have been described [25]. Thus, given the similarities of annelid and priapulid RAR sequences and ligand affinities, we anticipate an annelid-RAR-like structural interaction between ATRA and the priapulid RAR-LBP. A similar outcome is also expected for the RAR orthologues from nemertean, brachiopod and phoronid. Moreover, while in *Platynereis* ATRA displays a higher capacity to activate transcription via *RAR*, we find a similar pattern but for 9cisRA. Interestingly, 9cisRA was not detected in *Platynereis* tissues [25] and, at present, RA levels are unknown in priapulids. Furthermore, we did not explore the possibility of other endogenous and uncharacterized ligands to bind and activate transcription via RAR in priapulids, although this possibility should deserve future investigation. Overall, it remains a tantalizing question of the exact in vivo functions of PcauRAR and whether these are conserved between annelids and priapulids (and other protostomes). Additionally, the finding of RAR in Priapulida raises the interesting possibility that other RA signalling and metabolic components might be present in other protostome phyla such as Loricifera and Bryozoa. Future studies should be undertaken to firmly explore these hypotheses.

Finally, we examined whether PcauRAR can be exploited by EDCs by testing two organochloride pesticides, which have low water solubility, but are extremely persistent and particularly stable in soil [60]. Dieldrin was found in zooplankton and fishes from the Baltic Sea at concentrations between 15 and 170 ng/g lipid [55,56,58]; and <0.2–9.9 g and <0.15–0.8 g of dieldrin and endrin, respectively, were found per g of sludge and sediments [57]. Significantly, these compounds are known to disrupt the endocrine system in humans through the modulation of RA signalling pathways [34,54]. In agreement, with the study conducted in molluscs [24], we show that the tested pesticides were not able to activate PcauRAR and consequently induce gene transcription. To understand the mechanisms of action of EDCs in invertebrate lineages is problematic given the paucity of appropriate comparative approaches. For instance, of the various Ecdysozoa groups, only three (Insecta, Crustacea and Nematoda) have been thoroughly examined from an endocrinology standpoint [32]. Moreover, several aquatic pollutants have been reported to retard growth and moulting and influence mortality in crustaceans [61,62,63] and to affect growth, reproduction, life span and gene expression in nematodes [64,65]. Importantly, one of the clearest examples of endocrine disruption - imposex development in gastropods upon exposure to organotins, was shown to result from a specific interaction with the highly conserved NR *RXR* [66,67,68]. Moreover, the inclusion of comparative approaches and evolutionary thinking has highlighted the conserved and divergent biological responses to xenobiotics mediated by NRs (e.g., PPAR [69], PXR [70], ER [71]). Thus, defining the gene complement of NRs, their set of “natural” ligands and in vivo functions across the diversity of protostome phyla is fundamental to comprehend the impacts of EDCs in the Anthropocene epoch [72].

## 5. Conclusions

We provide here the first characterization of an Ecdysozoa RAR. Our findings, contribute to further clarify the early evolution of the RA gene module in Metazoa, supporting the hypothesis that RAR emerged as RA low-affinity sensor in the Bilateria, with the high-affinity RA binding profile acquired in chordates.

## Supplementary Materials

The following are available online at https://www.mdpi.com/2073-4425/10/12/985/s1, Video S1: Live specimens of *P. caudatus*; Figure S1: Analysis of the interaction between priapulid and human RAR-LBD-GAL4 with RXR-LBD-VP16 partner through a mammalian two-hybrid assay in COS-1 cells with no ligands. Table S1: List of primers used to isolate *P. caudatus RAR* and *RXR* genes, Table S2: List of sequences used for phylogenetic reconstruction of *TR*, *RAR*, *PPAR* and *RXR* genes and corresponding accession numbers, Table S3: List of primers used to amplify hinge region to LBD of RAR and RXR to be cloned into pBIND or pACT expression vectors.
