# Supplementary material for: An Orthologue of the Retinoic Acid Receptor (RAR) Is Present in the Ecdysozoa Phylum Priapulida
Source: Genes (Basel). 2019 Nov 29;10(12):985. doi: 10.3390/genes10120985 (PMC6947571; doi:10.3390/genes10120985)
Supplement: Supplementary file 1 [file genes-10-00985-s001.zip › Figure S1.docx]

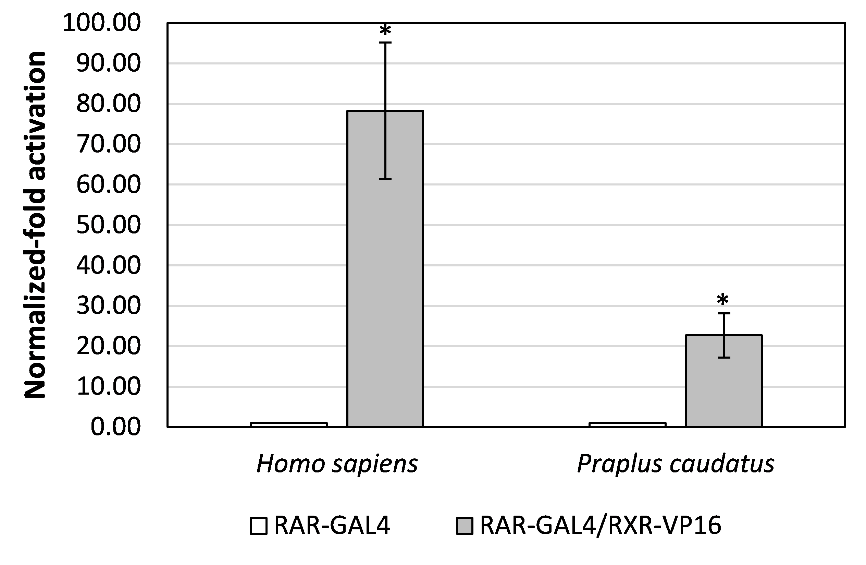


Supplementary Figure S1. Analysis of the interaction between priapulid and human RAR-LBD-GAL4 with RXR-LBD-VP16 partner through a mammalian two-hybrid assay in COS-1 cells with no ligands. Data represent normalized means ± SEM to the control from three separate experiments (n = 3). Cells transfected with no RXR-LBD-VP16 partner were used as control. Significant differences (* P<0.05) were inferred using one-way ANOVA.
